# A Dual Therapy of Nanostructured Lipid Carrier Loaded with Teriflunomide—A Dihydro-Orotate Dehydrogenase Inhibitor and an miR-155-Antagomir in Cuprizone-Induced C57BL/6J Mouse

**DOI:** 10.3390/pharmaceutics15041254

**Published:** 2023-04-17

**Authors:** Trideva Sastri Koduru, Vishal N. Gupta, Balamuralidhara Veeranna, Shanmuganathan Seetharaman

**Affiliations:** 1 Department of Pharmaceutics, JSS College of Pharmacy, JSS Academy of Higher Education & Research, Shivarathreeshwara Nagara, Mysuru 570015, India; 2 School of Pharmacy, Sri Balaji Vidyapeeth (Deemed to be University), Puducherry 607402, India

**Keywords:** teriflunomide, miR-155-antagomir, intranasal delivery route, cuprizone model, multiple sclerosis

## Abstract

The effective treatment of central nervous system (CNS) disorders such as multiple sclerosis (MS) has been challenging due to the limited ability of therapeutic agents to cross the blood–brain barrier (BBB). In this study, we investigated the potential of nanocarrier systems to deliver miR-155-antagomir-teriflunomide (TEF) dual therapy to the brain via intranasal (IN) administration to manage MS-associated neurodegeneration and demyelination. Our results showed that the combinatorial therapy of miR-155-antagomir and TEF loaded in nanostructured lipid carriers (NLCs) significantly increased brain concentration and improved targeting potential. The novelty of this study lies in the use of a combinatorial therapy approach of miR-155-antagomir and TEF loaded in NLCs. This is a significant finding, as the effective delivery of therapeutic molecules to the CNS has been a challenge in treating neurodegenerative disorders. Additionally, this study sheds light on the potential use of RNA-targeting therapies in personalized medicine, which could revolutionize the way CNS disorders are managed. Furthermore, our findings suggest that nanocarrier-loaded therapeutic agents have great potential for safe and economical delivery in treating CNS disorders. Our study provides novel insights into the effective delivery of therapeutic molecules via the IN route for managing neurodegenerative disorders. In particular, our results demonstrate the potential of delivering miRNA and TEF via the intranasal route using the NLC system. We also demonstrate that the long-term use of RNA-targeting therapies could be a promising tool in personalized medicine. Importantly, using a cuprizone-induced animal model, our study also investigated the effects of TEF-miR155-antagomir-loaded NLCs on demyelination and axonal damage. Following six weeks of treatment, the TEF-miR155-antagomir-loaded NLCs potentially lowered the demyelination and enhanced the bioavailability of the loaded therapeutic molecules. Our study is a paradigm shift in delivering miRNAs and TEF via the intranasal route and highlights the potential of this approach for managing neurodegenerative disorders. In conclusion, our study provides critical insights into the effective delivery of therapeutic molecules via the IN route for managing CNS disorders, and especially MS. Our findings have significant implications for the future development of nanocarrier-based therapies and personalized medicine. Our results provide a strong foundation for further studies and the potential to develop safe and economic therapeutics for CNS disorders.

## 1. Introduction

Multiple sclerosis (MS) is characterized by chronic disease conditions, including, mainly, the inflammation and demyelination of the healthy neurons in the central nervous system (CNS) [1]. Young adults are often affected by this neuron-disabling disease [2]. The clinical evidence established that MS disease pathogenesis is majorly affected by immune mechanisms; thus, it is considered an autoimmune disorder [3]. Research data have indicated that neuron attack in CNS is associated with the infiltration of T-lymphocytic subtype cells T-helper 17 cells (Th17) secreted by the interleukin 7 (IL-7) into the blood-brain barrier (BBB) [4]. This phenomenon leads to the inflammation and demyelination of neurons, thus depleting oligodendrocytes and, ultimately, neuronal degeneration. Unfortunately, treating MS is a challenging task with minimal effective therapies [5].

Teriflunomide (TEF) is an approved immunomodulatory molecule for treating relapsing–remitting MS [6]. TEF exercises its therapeutic potential by affecting the proliferation of impelled lymphocytes in MS [7]. Specifically, TEF reversibly inhibits the dihydro-orotate dehydrogenase—a mitochondrial enzyme expressed abundantly in proliferating lymphocytes—and thereby leads to the blockade of pyrimidine synthesis, thus disrupting the cell cycle and proliferating T- and B-cells to experience cytostatic activity, ultimately confining their influence in inflammatory mechanisms in MS [8,9].

Furthermore, significant evidence highlights miRNAs’ critical role in immunological pathways [10]. Interestingly, recent studies have exhibited numerous miRNA-associated immune responses in peripheral blood samples isolated from MS patients, consequently emphasizing the pivotal role of miRNAs in MS pathogenesis [11]. Several studies have established the vital role of miR-155 in regulating immune responses [12]. Research data have indicated its dysregulation. Specifically, overexpression is meticulously associated with affecting the permeability of the BBB, in addition to activating macrophages and T-immune cells, ultimately leading to the immune arbitrated damage of the myelin sheath, and thereby leading to neurodegeneration [13,14,15]. Furthermore, it has been established that miR-155 indirectly moderates the TGF-b signaling pathway by affecting SMAD2 and SMAD5. This further implies the significance of miR-155 and its role in MS disease pathogenesis [16]. miR-155 has been the most studied and explored miRNA, as it has been examined in numerous in vivo MS animal models. These studies implied the upregulation of miR-155 in CD4+ T-cells, thereby aiding the expression of inflammatory Th1 and Th17 cell subsets during disease induction [17]. Therefore, the inhibition of miR-155 expression would yield potential therapeutic benefits by preventing the processes involved in the pathophysiology of MS [18].

Conventionally, the patients suffering from neurodegenerative disorders are given oral drug administrations; however, the oral route is associated with poor bioavailability and yields poor therapeutic benefits [19]. Furthermore, oral drug administration poses several challenges and a poor ability to transcend the BBB: the therapeutic molecules are subjected to first-pass metabolism and enzyme degradation, which is associated with a faster clearance and a higher retention in peripheral tissues [20]. The advancements in drug delivery technologies have enabled research scientists to explore the potentials of intranasal (IN) administration, a non-invasive route of drug administration that potentially evades the BBB and enhances the bioavailability of therapeutic moieties [21]. The direct transport of drug molecules anticipated for higher CNS concentration is observed when delivered via the IN route and is supported by ample preclinical evidence [22]. When delivered via IN, the higher concentrations are achieved by following trigeminal, glymphatic, and olfactory pathways. There is abundant evidence suggesting the efficiency of IN administration with enhanced CNS concentration and better therapeutic benefit compared to other traditional delivery systems [23].

The application of nanocarriers have emerged as a promising strategy for drug delivery in recent years, offering significant advantages over traditional drug delivery methods. These carriers, including liposomes, polymeric nanoparticles, exosomes, and extracellular vesicles (EVs), can be designed to encapsulate and deliver drugs to specific cells or tissues, minimizing off-target effects and reducing toxicity. The use of nanocarriers in neurodegenerative diseases and brain disorders shows great potential for delivering drugs that target specific pathological mechanisms involved in these diseases. Recent scientific papers have explored various approaches to optimize the functionality and specificity of nanocarriers. The peptide-based functionalization of NDS showed significant advantages, including the augmentation of the ability of nano delivery systems (NDS) to target specific receptors or mutant proteins on the surface of cancer cells [24]. The peptide-based functionalization with EVs paved the way for the conjugation of biological NDS with receptor-targeted peptides for cancer therapy and diagnosis. Additionally, the surface functionalization of EVs has shown promise in achieving better targetability and specificity for drug delivery [25].

Overall, the use of nanocarriers, including exosomes and EVs, has the potential to revolutionize drug delivery in treating various diseases. However, further research is needed to fully understand the mechanisms involved in nanocarrier-mediated drug delivery and optimize their use in clinical settings. Recent studies have shown promising results, including developing the first Aβ-targeted ratiometric H_2_O_2_-responsive fluorescent probe for the real-time detection and monitoring of the Aβ-induced H_2_O_2_ level in cell and AD mouse models [26].

Nanocarriers represent an exciting area of research with the potential to significantly improve the efficacy and safety of drug delivery for a wide range of diseases. Developing more effective and specific nanocarriers holds promise for treating neurodegenerative diseases and brain disorders. The developments in novel drug delivery systems have enabled formulation scientists to explore the potential of nanocarrier systems to achieve better therapeutic benefits [27]. These innovations led to investigating IN delivery by encapsulating the therapeutic moieties in these carrier systems [28]. However, only suitable release kinetics with the optimum dose and readiness of drug molecules at the absorption site would lead to maximum benefits [19]. Nanocarrier systems with biocompatible polymers possessing an amphiphilic nature are beneficial for therapeutic loading molecules. Fascinatingly, these nanocarriers inherently are versatile and enable the researchers to modify them according to the research interest that ultimately facilitates efficient delivery [29,30]. Polymeric, lipid, metallic, and non-metallic nanocarriers have been extensively explored and studied for their potential to transcend the BBB [31]. These formulation approaches could incite tremendous enthusiasm in various research societies to explore and study their potential for IN administration to alleviate numerous neurodegeneration conditions [32].

Our study hypothesizes that the developed NLCs loaded with miR-155–antagomir and TEF can effectively manage MS-associated neurodegeneration and demyelination by improving drug bioavailability and brain targeting potential. This study aims to develop and evaluate the pharmacokinetic parameters, efficacy, and safety of the developed NLCs for the management of MS. The findings of this study can pave the way for further research in the effective delivery of therapeutic molecules via the intranasal route for managing neurodegenerative disorders. These nanocarriers easily bypass the BBB via the intranasal route, and adequate drug levels could be achieved at a site of action. Therefore, nanocarrier-loaded therapeutic agents are significant for therapeutics’ safe and economical delivery in treating CNS disorders [33]. As there is no abundant literature on the effective delivery of miRNAs via intranasal delivery, the findings of this study remain instrumental for further studies and for the effective delivery of therapeutic molecules via the intranasal route (non-invasive) to the CNS to manage neurodegenerative disorders.

## 2. Materials and Methods

### 2.1. Chemicals

Teriflunomide was obtained from Shilpa Medicare (Bangalore, India), miR-155-antagomir (5′-ACCCCUAUCACGAUUAGCAUUAA-3′) was purchased from Synbio Technologies (Monmouth Junction, NJ, USA), and precirol and maisine were obtained as gift samples from Gattefosse India (Mumbai, Maharashtra, India). Cuprizone and rhodamine B were obtained from Sigma (Chennai, Tamil Nadu, India). Luxol Fast Blue, Hematoxylin, and eosin stain were obtained from HiMedia (Hyderabad, Telangana, India) and Merk (Mumbai, Maharashtra, India). Glial fibrillary acidic protein (GFAP) was obtained from Master Diagnostica (New Delhi, Delhi, India), anti-proteolipid protein (PLP) was obtained from Novus Biologicals and MAC-3 was obtained from Abcam (New Delhi, Delhi, India). All of the chemicals used in the study had a purity level greater than 95%.

### 2.2. Mouse

Female eight-to-ten-week-old C57BL/6 mice were obtained from Adita Biosys Pvt Ltd. (Tumkuru, Karnataka, India) with a mean weight of 35.86 g ± 1.31 (SD). Mice were housed as required in clean cages under standard laboratory conditions. Sterilized bedding, food, and portable mineral water were available ad libitum. The experiments were conducted in strict accordance to the Institutional Animal Ethics Committee (Approval No. JSSAHER/CPT/IAEC/015/2020).

### 2.3. Formulation of TEF–miR-155-Antagomir-Loaded NLCs

Nanostructured lipid carrier systems (NLCs) have emerged as promising drug delivery platforms due to their high drug-loading capacity and improved bioavailability. To prepare NLCs, various methods have been developed, including hot homogenization and microfluidic-based techniques [34].

The hot homogenization method has been widely used by several researchers to prepare NLCs. This method involves mixing the lipid and aqueous phases at a specific temperature under stirring conditions to obtain a pre-emulsion. The lipid phase consists of a combination of solid and liquid lipids, along with surfactants and other additives to enhance the stability and drug-loading capacity of the NLCs. The pre-emulsion is then homogenized using an ultrasonic or mechanical homogenizer to form the final NLCs.

For instance, Zhao et al. prepared ultra-small NLCs (usNLCs) with a particle size of 25 nm using the solvent diffusion method. The researchers used a combination of Glycerin monostearate (GM), phosphatidylcholine (PC), oleic acid (OA), and stearic acid-polyethene glycol-folate (SA-PEG-FA) to encapsulate paclitaxel. The study results showed that the PTX-loaded usNLC exhibited superior in vitro and in vivo performances compared to Taxol [35]. Similarly, Ghani et al. used the hot homogenization method to prepare NLCs for the transdermal delivery of Ficus deltoidea extracts. The authors used stearic acid (SA) as the solid lipid and oleic acid (OA) as the liquid lipid, along with poly(vinyl alcohol) (PVA) as a stabilizer. The DESD technique was found to exhibit superior performance for encapsulation [36].

**Figure 1:**
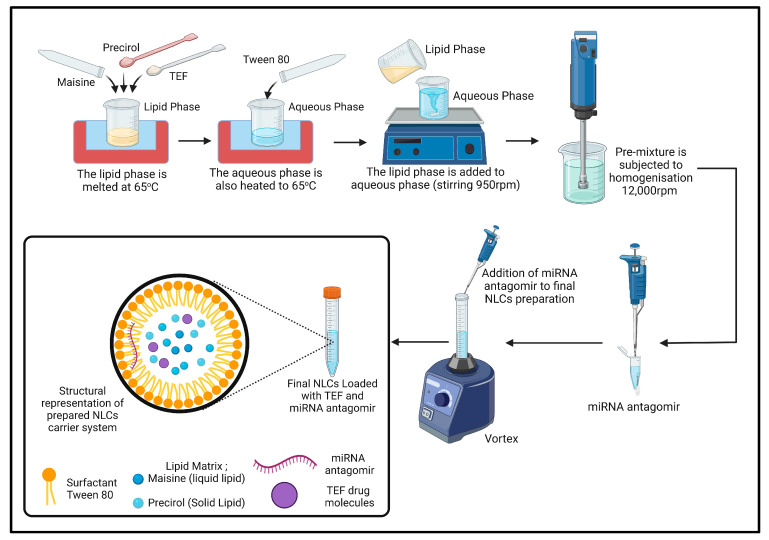
Schematic representation highlighting the steps involved in the preparation of NLCs along with its structural picture.

In our research, we used a different combination of lipids and surfactants and a homogenization method to prepare NLCs. A pre-emulsion is first obtained by mixing the lipid and aqueous phases at a temperature of 65 °C under stirring condition (950 rpm). The lipid phase, comprising 1.5% (*w*/*w*) precirol, 0.75% (*w*/*w*) maisine, and precisely 0.1% (*w*/*w*) TEF, was added to the lipid phase in its melted state. Subsequently, the aqueous phase comprising 2.25% (*w*/*w*) tween 80 was added in DEPC water. The final NLC mass was 20 g. Following the formation of pre-emulsion, it was subjected to homogenization (IKA T25 Ultra Turrax, Bengaluru, India) at 12,000 rpm for 10 min. Following homogenization, the miR-155-antagomir (10 nM final concentration) was accurately applied to the final formulation and incubated for 40 min at room (Figure 1).

The prepared formulation were characterized by their particle size, polydispersity index (PDI), and zeta potential by (Malvern Instruments, Malvern, UK). The NLC formulations were suitably diluted (1:100 *v*/*v*) in particle free millipore water and measured in a cuvette (polystyrene) with a path length of 10 mm at 25 °C [37].

The centrifugation method was employed for the estimation of encapsulation efficiency (EE) of TEF in developed NLCs [38]. An amount of 2.0 mL of TEF-loaded NLC was subjected to centrifugation (REMI cooling ultracentrifuge, Mumbai, India) at 12,000 rpm for 25 min. During the centrifugation process, the temperature was maintained at 4 °C. Then, the supernatant was carefully separated and suitably diluted with methanol to determine the free drug. The diluted samples were analyzed by LC-MS. The EE was estimated using Equation (1);

(1) $$\mathrm{EE}=\frac{\left(\mathrm{total}\;\mathrm{amount}\;\mathrm{of}\;\mathrm{TEF}\;\mathrm{used}\;-\;\mathrm{free}\;\mathrm{TEF}\;\mathrm{in}\;\mathrm{supernatant}\right)}{\mathrm{total}\;\mathrm{amount}\;\mathrm{of}\;\mathrm{TEF}\;\mathrm{used}}\;\times \;100\;$$

### 2.4. Cellular Uptake

The cellular uptake of nanocarriers is a crucial aspect to consider for developing effective drug delivery systems. In our study, we aimed to understand the uptake of rhodamine-tagged NLCs by cells. We seeded 2 *×* 10^5^ cells in a 60 mm plate and added approximately 5 µL of rhodamine-tagged NLCs loaded with TEF–miR-155–antagomir. We captured images using a fluorescent microscope equipped with red and blue filters for the rhodamine and Hoechst stain, respectively, and phase contrast images of the same field. By merging these images using ImageJ software, we were able to visualize the uptake of NLCs by the cells at different time intervals. Understanding the uptake mechanism of nanoparticles is essential for designing and optimizing drug delivery systems. Overall, our study provides valuable insights into the uptake mechanism of cells for rhodamine-tagged NLCs. This understanding can inform the development of novel nanocarriers for drug delivery applications, leading to more effective and targeted therapies. The RAW 264.7 cells were employed for this study.

### 2.5. Cuprizone Treatment

MS is strongly associated with the demyelination of the CNS. Over the years, cuprizone-induced toxicity has been comprehensively investigated to study demyelinating disorders [39]. Bis(cyclohexanone)oxaldihydrazone, otherwise known as cuprizone, is fundamentally a copper–chelating agent that potentially stimulates reproducible demyelination in mice [40]. Hence, in our study, the induction of the demyelination was achieved by oral administration of cuprizone. In most cuprizone models, the mice are usually given cuprizone-containing pellets or a powder diet mixed with cuprizone; however, these approaches were challenging for researchers since there was no confidence in the homogenous mixing of cuprizone via the diet [41]. Furthermore, there was no way to control the level of intake, resulting in ambiguity in dose administration. Another primary concern was that cuprizone is very sensitive to the environment, and may degrade following long-term environmental exposure. All these factors together constitute discrepancies in the dose, thereby resulting in different variations of demyelination in each mouse [42]. We considered direct oral administration to avoid these drawbacks of diet administration. Cuprizone was added to 1% methylcellulose and vortexed to achieve homogeneous cuprizone-methylcellulose suspension. In brief, the mice were administered doses of 400 mg/kg/day by gavage with 10 mL/kg volume. The suspension was freshly prepared prior to dose administration. The induction was carried out for a period of six weeks.

### 2.6. Study Design and Treatment Protocol

#### 2.6.1. For Pharmacokinetic Study

TEF Suspension (free drug): this group received the IN administration of TEF suspension in which the TEF was freely dispersed in saline solution.

TEF NLC Intranasal: this group received the IN administration of developed TEF-loaded NLCs.

TEF NLC Intravenous: this group received the intravenous (IV) administration of TEF-loaded NLCs.

○ Note: Each mouse received equal doses equivalent to 5 mg/kg dose.

#### 2.6.2. For Pharmacodynamic Study Mice Were Randomly Allocated to Four Groups as Mentioned below

- Normal Group: this group of animals received food and potable mineral water ad libitum. Furthermore, no chemical was administrated. This group remained the control group throughout the experimentation (*n* = 6).
- Disease Control Group: the demyelination was induced by the oral administration of 400 mg/kg/day of cuprizone by oral gavage. This group received no further treatment (*n* = 8).
- TEF–Oral Group: The demyelination was induced by the oral administration of 400 mg/kg/day of cuprizone by gavage. Furthermore, the group simultaneously received oral gavage of TEF suspension at a dose of 5 mg/kg/day for six weeks (*n* = 8).
- TEF–IN Group (NLCs loaded with dual TEF–miR-155-antagomir): The demyelination was induced by the oral administration of 400 mg/kg/day of cuprizone by gavage. Furthermore, the group simultaneously received TEF–miR-155-antagomir-loaded NLCs with doses of 5 mg/kg/day of TEF and 10 nmol of miR-155-antagomir intranasally. For IN administration, the mice were held and 30μL of TEF–miR-155-antagomir NLC preparation was administered.

### 2.7. LC-MS Specifications and Mobile Phase

The drug quantifications were analyzed by LC-MS [43]. The analysis used UPLC–MS (Acquity H-Class, Waters Corporation, Milford, MA, USA). An integrated vacuum degasser and an ultra-performance binary solvent manager were also used. The instrument was attached to the C18 column (2.1 × 50 mm, 1.7 mm) with a gradient flow pump with autosampler. The study’s mobile phase comprised 0.1% formic acid (A) and acetonitrile (B). The elution was gradient with a constant flow rate of 0.6 mL/min with an injection volume of 5 mL. During the complete analysis, the column was maintained at ambient temperature. The MS conditions consisted of a positive polarity electrospray ionization (ESCi) source; a probe temperature of 450 °C; a sampling cone voltage of 30 V; a source temperature of 150 °C; a source offset voltage of 80 V; a sample infusion flow rate of 5 mL/min, a collision energy ramp of 6 eV (argon, collision gas); and a mass ranging from 50 to 1500 *m*/*z*. All of these constitute the acquisition parameters. Furthermore, all acquired data were processed using waters corporation mass lynx software (V4.1, Milford, MA, USA).

### 2.8. Pharmacokinetic Parameters

The pharmacokinetic investigations were performed to understand the pharmacokinetics parameters of TEF. The blood samples were collected by the retro-orbital puncture method, which involved the collection of blood from the orbital sinus of anaesthetized mice. The samples were collected at predetermined intervals of 2, 4, 6, 8, and 24 h, and a quantity of 0.5 mL was collected from assigned groups at each time point. The collected blood samples were carefully transferred to labeled, precoated EDTA tubes for further estimations.

The collected samples were centrifugated at 8000 rpm for 15 min to isolate the plasma using a REMI cooling centrifuge. The plasma was then carefully separated and extracted for TEF using a liquid–liquid extraction approach. Specifically, 0.3 mL of LCMS-grade methanol was added to the plasma sample, which was then vortexed for 20 min. The sample was then centrifuged again for 10 min at 6000 rpm to facilitate the complete extraction of TEF into methanol. Finally, the concentration of TEF in the supernatant was estimated using LC-MS, a highly sensitive and accurate analytical technique.

In addition to plasma, the concentration of TEF in the brain was also examined. To this end, mice were sacrificed by cervical dislocation at predetermined intervals after blood collection. The brains were carefully isolated and thoroughly cleaned using saline phosphate buffer to eliminate adherent tissues. The brains were then homogenized with methanol in a tissue homogenizer (FastPrep-24TM Classic high-speed homogenizer) to obtain a clear supernatant. Any remaining tissue debris was removed by centrifugation, and the supernatant was examined for TEF concentration using LC-MS. To determine the pharmacokinetic parameters of TEF, the concentration of TEF in both plasma and brain was plotted against time intervals. Non-compartment modelling was used to calculate the maximum concentration (Cmax), the time at which maximum concentration occurs (Tmax), and the area under the curve (AUC). These parameters provide critical information about the behavior of TEF in the body and its potential therapeutic applications. Overall, the experimental procedures were meticulously carried out, and the results obtained provide valuable insights into the pharmacokinetics of TEF [44].

#### Determination of Targeting Efficiency

The targeting efficiency of the nanoparticles and drug suspension following intranasal administration can be equated by brain-to-plasma concentration, which is explicated as partition coefficient (*Kp*) and calculated by the ratio of C_brain_/C_plasma_. The high brain targeting is indicated by high *Kp* after an intranasal administration [45,46]. The equations employed for calculating the targeting efficiency, including those for drug targeting efficiency (DTE), direct transport percentage (DTP), and drug targeting index (DTI), are as follows [47,48]:

(2) % DTE =AUC0−24 brain INAUC0−24 blood INAUC0−24 brain IVAUC0−24 blood IV  × 100 

(3) $$\%\;\mathrm{DTP}=\frac{AUC0-24\left(brain\right)IN\;-\;\left(\frac{AUC0-24\;\left(brain\right)IV}{AUC0-24\;\left(blood\right)IV}\right.\;\times \;\left.AUC0-24\;\left(blood\right)IN\right)}{AUC0-24\;\left(brain\right)IN}\;\times 100$$

(4) $$\mathrm{DTI}\;=\frac{\left[AUC\right.\;\left(brain\right)/AUC\;\left.\left(Blood\right)\right]\;IN}{\left[AUC\right.\;\left(brain\right)/AUC\;\left.\left(Blood\right)\right]\;IV}$$

(5) $$\mathrm{Absolute}\;\mathrm{bioavailability}\;=\frac{AUC\;\left(brain\right)\;IN\;\left(NLCs\;Formulation\right)}{AUC\;\left(brain\right)\;IV\;\left(NLCs\;Formulation\right)}\times 100$$

(6) $$\mathrm{Relative}\;\mathrm{bioavailability}\;=\frac{AUC\;\left(brain\right)\;IN\;\left(NLCs\;Formulation\right)}{AUC\;\left(brain\right)\;IN\;\left(Drug\;Suspension\right)}\times 100$$

### 2.9. Histopathology and Immunohistochemistry (IHC)

The study was conducted on a cohort of male mice randomly assigned to different groups. Prior to the study induction, the mice were acclimated to the laboratory environment for one week to minimize stress. The study protocol was approved by the Institutional Animal Ethics Committee (IAEC) and was conducted in accordance with the guidelines established by the Committee for the Purpose of Control and Supervision of Experiments on Animals (CPCSEA), Government of India for the care and use of laboratory animals.

The mice were carefully prepared for analysis, beginning with administering an anesthetic using a combination of ketamine (Aneket^®^, New Delhi, Delhi, India) and xylazine (Xylaxin^®^, New Delhi, Delhi, India) to ensure minimal discomfort. After a period of six weeks, the groups were euthanized by decapitation to obtain the brains for further examination. Each brain was meticulously dissected and immersed in 4% formaldehyde to preserve the tissue until the analysis could begin. The analysis focused on 4–8 mm coronal sections from the bregma region of the brain. To ensure the accuracy of the analysis, the tissue processing and staining were performed by a single researcher who was trained and experienced in the techniques used. The LFB staining was used to visualize the myelin sheaths, which appear as blue-stained areas in the tissue sections. Hematoxylin and eosin staining was used to detect any signs of inflammation, which appear as areas of redness or swelling in the tissue sections [49,50].

The prepared slides were viewed under the LX-500 LED trinocular research microscope (Labomed), and images were captured using MiaCam CMOS. The semi-quantitative scoring scale used to assess the level of myelin loss and inflammation was based on established criteria in the literature. The two observers who performed the scoring were also trained and experienced in the analysis of brain tissue. To ensure consistency between the observers, a subset of the tissue sections was randomly selected and scored independently by each observer. The inter-rater reliability was assessed using the intraclass correlation coefficient (ICC), which indicated excellent agreement between the observers (ICC > 0.90) [51,52].

The study used brain tissue samples from normal healthy and cuprizone-treated groups to investigate myelin, astrocytes, and macrophages/microglia immunostaining changes. Tissue samples were collected from both groups at specific time points and processed for staining using primary antibodies against myelin–PLP (1:150 dilution), astrocytes–GFAP (1:150 dilution), and macrophages/microglia–Type 1 membrane glycoprotein, MAC-3 (1:1500). The brain tissue sections were initially deparaffinized in xylene for five minutes and rehydrated with alcohol (100% for eight minutes, followed by 90% and 80% for six minutes each). Peroxidase blocking was achieved using 3% hydrogen peroxide, followed by antigen retrieval performed by microwaving the sections in Tris-EDTA buffer (pH 9.0). After thorough rinsing with distilled water and phosphate buffer saline, the sections were blocked with bovine serum albumin (0.25%) for thirty minutes at room temperature.

The sections were then incubated overnight at 40 °C with primary antibodies (GFAP, PLP, and MAC-3) at the proper dilution. After rinsing with phosphate buffer saline, the sections were covered with a secondary antibody (anti-mouse antibody–IgG conjugated with HRP-Conjugate). Prepared slides were then covered with a readymade 3,3′-diaminobenzidine (DAB) substrate buffer containing DAB chromogen. After approximately five minutes, the slides were gently rinsed in running water and counterstained with hematoxylin. The excess was gently washed with running water and differentiated with acid alcohol (1%). Finally, the prepared sections were dehydrated with alcohol, cleared with xylene, and mounted with DPX. Data were collected and analyzed to evaluate differences in myelin, astrocytes, and macrophages/microglia immunostaining between the two groups. The study employed careful and precise staining procedures to ensure accurate results for the immunostaining investigation. Normal healthy and cuprizone-treated groups of brain tissue served as controls for all of the staining. The study’s findings provide valuable insights into the pathological processes associated with demyelination and inflammation in the central nervous system and can lead to further advancements in diagnosing and treating related disorders such as multiple sclerosis.

In addition to the staining procedures described earlier, the study utilized a Zeiss Axioplan 2, (Carl Zeiss, Göttingen, Germany) high-quality light, to analyze the prepared sections. The reactive astrocytosis, indicated by GFAP immunoreactivity, was evaluated using a semi-quantitative system in which a score of 0 represented no immunoreactivity, and a score of 3 corresponded to extensive immunoreactivity. An observer blinded to the treatment groups observed immunopositive cells using an ocular morphometric grid to evaluate activated macrophages and microglia. Digital densitometry was employed to quantify the immunopositivity of PLP, and the area of immunopositivity related to the percentage of the total image area was considered to express the immunopositivity.

The sections were evaluated in four brain regions, including the corpus callosum (CC), caudate putamen (CP), cerebral cortex (CE), and hippocampus (HC). These regions were chosen based on their involvement in developing and progressing demyelinating and inflammatory diseases in the central nervous system. Using the Zeiss Axioplan 2 microscope and the various quantification methods employed allowed for accurate and precise evaluation of the staining results and provided valuable data for investigating the pathological processes associated with demyelination and inflammation in the central nervous system.

### 2.10. Statistical

For the present research, the statistical analysis was conducted using the GraphPad Prism software (version 9.5.0). Specifically, the data were analyzed using a combination of one-way analysis of variance (ANOVA) and the Wilcoxon test. The one-way ANOVA is a parametric statistical method that tests whether statistically significant differences exist between the means of two or more groups. It is commonly used in research to compare the means of several groups with a single factor. In this case, one-way ANOVA was chosen as it is suitable for analyzing data with multiple groups. Following the ANOVA, a Wilcoxon test was employed to assess whether there was a statistically significant difference between the two paired groups. The Wilcoxon test is a non-parametric statistical method used to compare paired data in which the differences between the two groups are not normally distributed. In order to determine whether the results were statistically significant, a significance level (α) of *p* < 0.05 was set. This indicates a 5% probability of obtaining the observed results by chance alone. The results were deemed statistically significant if the *p*-value was less than 0.05.

## 3. Results and Discussions

### 3.1. Formulation of TEF–miR-155-Antagomir-Loaded NLCs

**Figure 2:**
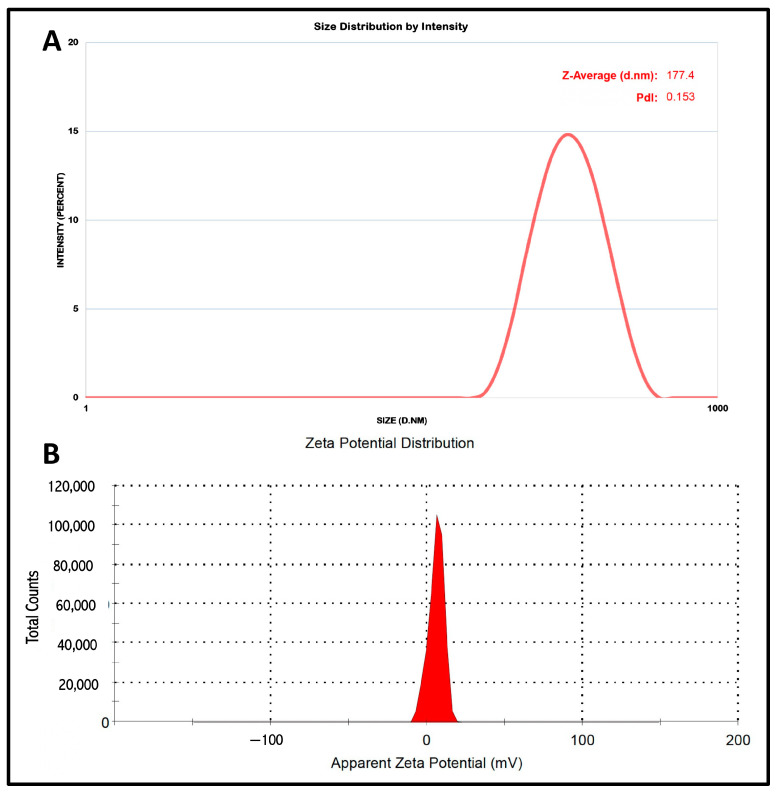
NLC system characterization. (**A**) Particle size: 177.4 nm; (**B**) zeta potential: 5.95 mV.

Our study comprised the development of an NLC system for intranasal delivery. For the effective intranasal administration of NLCs and, consequently, delivery via the nose to the brain, the particle size and the PDI remain very vital. The studies indicated that to achieve effectual brain targeting a particle size below 200 nm is desired. The developed nanocarrier system were characterized by particle size and PDI and measured by (Malvern Instruments, Morvern, UK). The particle size and the PDI of the developed NLC system were determined to be 177.4 nm and 0.153, respectively. The small PDI indicates the uniform distribution of NLC particles, which enables constant drug release via the polydisperse system. The NLC system, owing to its fine particles, possesses a larger surface area, and consequently enhances the release rate of the TEF. The stability of the particulate suspension system relies on the electrostatic repulsion among the dispersed particles in the suspending system, which are also responsible for the interaction with the cell membrane. The Zeta potential indicated the electrostatic repulsion of the lipid carriers in the NLC system. The droplet surface charge of the NLC system was estimated by (Malvern Instruments, UK). The zeta potential of the formulation was recorded as 5.95 mV. The results of the zeta potential suggest the stability of the NLC system (Figure 2).

An accurate EE is a significant aspect for achieving anticipated drug concentration at the site of absorption. Usually, the EE is articulated as a percentage and is signified by the ratio of the actual amount of TEF entrapped in the prepared NLCs to the actual amount of TEF added for the preparation of NLCs. Higher amounts of entrapment are always expected as this results in better drug release at the site of absorption. The EE was calculated by Equation (1), and the results indicated good EE amounting to 93.06 ± 3.34%.

### 3.2. LCMS Linearity

**Figure 3:**
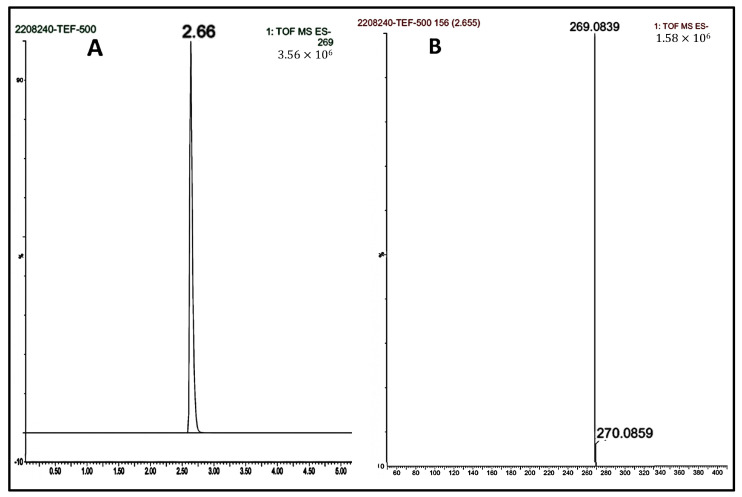

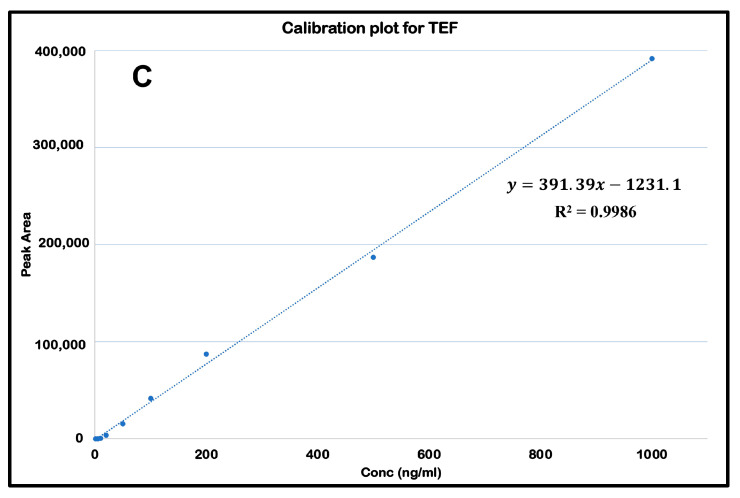
UPLC-MS TEF chromatograms. (**A**) Chromatogram for 500 ng/mL standard concentration with a retention time of 2.647 min. (**B**) Chromatogram obtained from the analysis of TEF representing m/z values. (**C**) Calibration curve of TEF in triplicates.

Initially blank plasma samples were prepared from the samples collected from negative control groups to ascertain no potential interferences by any endogenous plasma compounds. Further plasma and tissue homogenization calibration curves entailed nine concentrations ranging from 1.0 to 1000.00 ng/mL. The calibration curves were constructed by plotting the peak area of the standard concentration against their respective concentrations. The R^2^ was 0.998, calculated with the equation y = 391.39x − 1231.1. The retention time was determined to be 2.647 min. The mass selective detector (MSD) was fixated at a positive ionization mode with selected ion monitoring. The concentrations of the unknown samples were determined from the interpolation from the calibration curve (Figure 3).

### 3.3. Cellular Uptake

**Figure 4:**
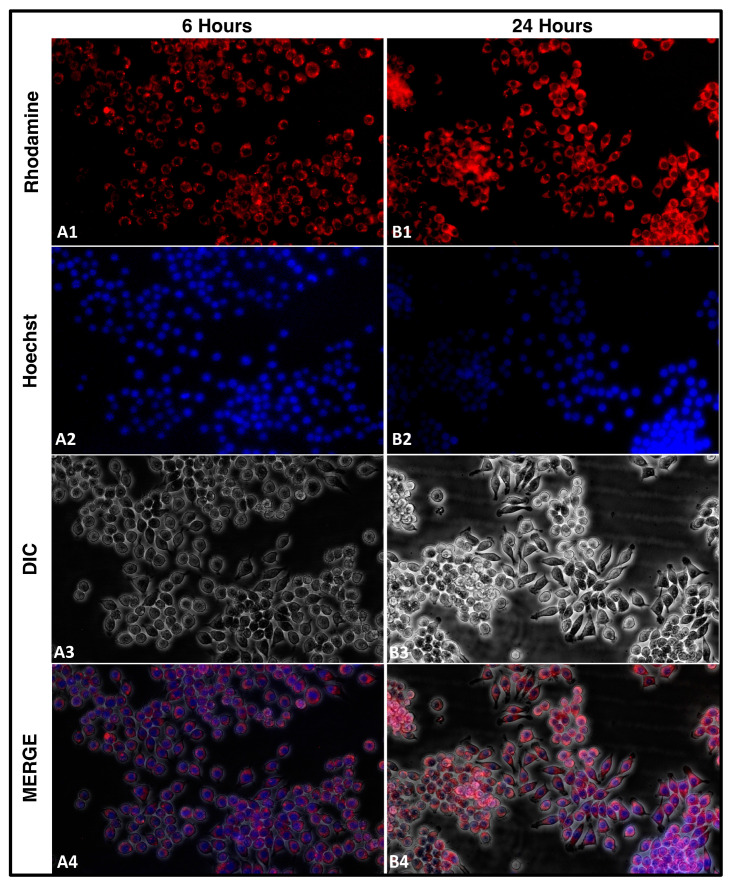
Cellular uptake of the rhodamine-tagged NLCs. (**A**) represents images captured at 6 h, while (**B**) represents images captured at 24 h. (**A1**) Indicates the Rh-NLCs intensity after 6 h. (**B1**) Increased intensity of Rh-NLCs at 24 h increased higher uptake of Rh-NLCs. Similarly (**A2**,**B2**,**A3**,**B3**) indicate the uptake of Rh-NLCs at 6 h and 24 h respectively, the images signify high uptake at 24 h. (**A4**) Merged image indicating uptake at 6 h, subsequently the (**B4**) Merged image with the increased intensity signifying high uptake of the Rh-NLCs at 24 h. (Magnification 10×).

The efficient cellular uptake of drugs from their delivery vehicles is crucial for achieving the desired therapeutic effects. To study the cellular uptake of NLCs loaded with TEF-miR-155-antagomir, these particles were chemically tagged with rhodamine B, a contrast agent commonly used for fluorescence imaging. In order to understand the effect of these particles on cells, it is vital to determine their ability to permeate the cell membrane and reach the cytoplasm. Therefore, the rhodamine-tagged NLCs were evaluated for their cellular uptake potential. The results showed that the Rh-NLCs could penetrate the cell membrane and enter the cytoplasm. The fluorescence images of the cells revealed a gradual increase in the amount of fluorescence over time with a significant increase observed at 24 h. The fluorescence was distributed uniformly throughout the cytoplasm of the cells (Figure 4).

The ability of these particles to efficiently enter the cytoplasm of cells is an essential factor in their potential therapeutic applications. The fluorescent imaging technique used in this study provides a valuable tool for evaluating the efficacy of drug delivery systems and monitoring the NLCs intracellular fate. Overall, this study highlights the potential of NLCs as drug carriers and the importance of understanding the cellular uptake mechanisms of these carriers for optimizing treatment efficacy.

### 3.4. In Vivo Pharmacokinetic Study

Intranasal drug delivery is a progressing, reliable, and promising pathway to deliver an inclusive range of therapeutic molecules to the central nervous system (CNS) for the treatment of numerous brain diseases. This delivery system offers a non-invasive entry into the brain via direct nose-to-brain and/or indirect nose-to-blood-to-brain routes. In our study, the TEF concentration in the brain was estimated following the intranasal administration of TEF-loaded NLCs in addition to TEF suspension and the intravenous administration of TEF-loaded NLCs. The TEF plasma concentrations and brain concentrations against predetermined time intervals were plotted for each. The pharmacokinetic parameters, including Cmax, Tmax, AUC, and the area under the moment curve (AUMC), and the neuropharmacokinetic parameters, including DTI, DTP, and DTE, were calculated using Equations (2) to (6).

**Figure 5:**
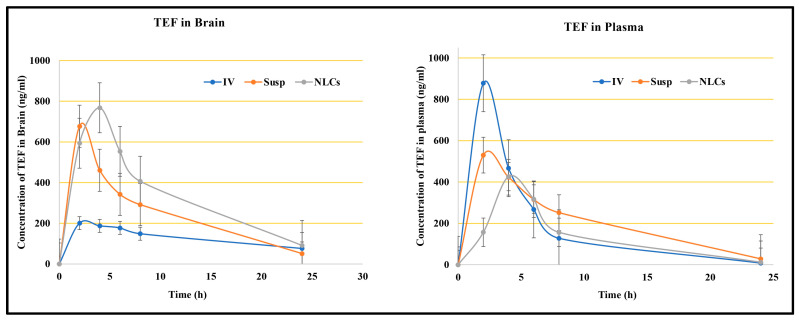
The brain and plasma drug profiles after intravenous (IV) and intranasal (IN) administration of both NLCs and free TEF suspension. The study was performed in triplicate data that are shown in mean ± SD (*n* = 3); the statistical analysis was performed between each group and the differences were considered significant (*p* < 0.005). The IN administration of NLC system was highly significant (*p* < 0.002) to TEF free suspension group.

For a better comparison, the administered dose of TEF was kept constant. Figure 5 reveals the plasma and brain levels of TEF from NLCs and from the free drug resulting from intranasal and intravenous administration. The TEF plasma profile indicates the higher concentration of TEF following the intravenous administration of TEF-loaded NLCs in comparison to other formulations at 2 h. The intranasal administration of TEF-loaded NLCs yielded the highest plasma concentration only at 4 h. Conversely, there was no significant distinction in TEF plasma concentrations after 5 h. Fascinatingly, TEF plasma concentrations of both TEF-loaded NLCs administered intranasally and intravenously remained higher than that of the free drug administration. It is understood that the delay in the peak concentration for intranasal administration is due to the movement of drug molecules across the nasal membrane; however, in intravenous administrations, the drug readily reaches the circulating blood, and, hence, a faster peak concentration is observed. The diffusion of TEF via the polymer matrix leads to a significant concentration variation among the free drug and NLC systems.

Similarly, the brain samples indicate a higher TEF concentration at 2 h for intranasally administered TEF suspension; however, by 4 h TEF-loaded NLCs administered intranasally resulted in a higher TEF concentration in the brain compared to the free drug. The TEF brain levels from TEF-loaded NLCs were higher compared to the other formulations in the study. This suggests improved and enhanced permeation through the nasal mucosa. Furthermore, the lipophilicity of the developed NLCs indicates a better inhibition of drug efflux. The pharmacokinetic data suggest enhanced brain efficiency following the intranasal administration of TEF-loaded NLCs. The pharmacokinetic parameters were calculated for blood and brain samples and are indicated in Table 1.

Additionally, our study considered both TEF free suspension and TEF-loaded NLC administrations to estimate the transportation of TEF to the brain. The DTE and DTP values were calculated. The intranasal administration of TEF-loaded NLCs exhibited higher DTP and DTE values in comparison to TEF free suspension. Usually, the drug molecules administered intranasally effectively reach the brain by evading the BBB, which is a phenomenon established by DTP. The drug transport is attained through the olfactory bulb. For TEF-loaded NLCs, the DTP value was determined to be 72.03% compared to that of TEF free drug suspension, i.e., 41.72%, when administered intranasally. Furthermore, a higher value of DTE, 357.55%, was determined after the TEF-loaded NLCs enhanced the brain targeting efficiency for the developed NLC system. Similarly, DTI was determined to be 3.58%, with (DTI > 1) signifying effective brain delivery. It is understood that higher DTP and DTE values indicate the direct delivery of drug molecules from the nose to the brain, which is mainly conducted via the olfactory and trigeminal pathway. Hence, the higher DTP and DTE values of the developed NLC systems, compared to the free drug suspension, suggest a higher permeability of TEF drug molecules to the brain. In addition, the drug encapsulation facilitates protection against significant degradation and helps evade efflux mechanisms.

### 3.5. In Vivo Pharmacodynamic Study

In neurodegenerative disorders, the potential harm caused to the CNS is treated by employing disease-modifying treatments. MS is a potentially chronic and progressive disorder often portraying symptoms of demyelinating conditions. Several kinds of research indicate that immune mechanisms affect significant CNS deterioration. Usually, the patients are often observed with copious perivascular inflammatory centers; these remain the epicenter for further demyelination and other neuronal injuries. Furthermore, these lesions attract the infiltration of numerous immune cells and T-cells that directly affect neuronal cells. There is a prominence of aberrant immune activity in MS disease pathogenesis, and the disease-modifying therapies should notably restrict the MS-linked immune activity to yield therapeutic significance. Conversely, these therapies should not interfere with regular immune mechanisms towards numerous pathogens, and should instead exhibit minimal effects on protective immune mechanisms.

The potential role of miRNAs in regulating and modulating critical pathways for disease progression has attracted us to develop novel RNA-based therapy for managing MS. Our study emphasizes the importance of developing an integrated dual therapy exploring an intranasal route that comprises an miR-155-antagomir, in combination with teriflunomide, thus targeting the brain via NLCs systems in cuprizone-induced C57BL/6J.

#### Histopathology and IHC

**Figure 6:**
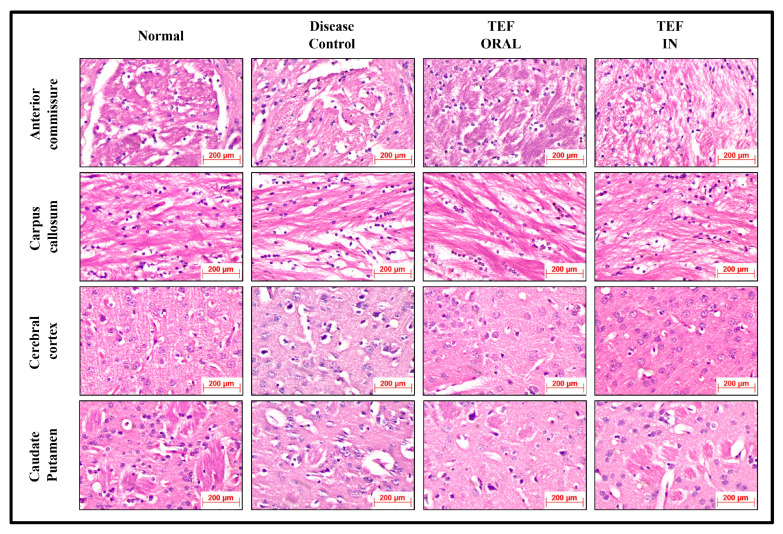
The H&E-stained coronal sections of the Normal, Disease Control, TEF oral, and TEF IN treatment groups (400×).

**Figure 7:**
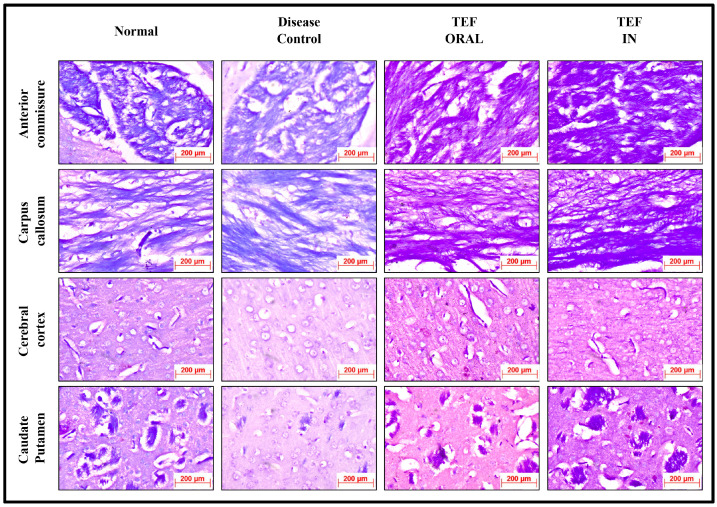
The LFB-stained coronal sections of the Normal, Disease Control, TEF oral, and TEF IN treatment groups (400×).

**Figure 8:**
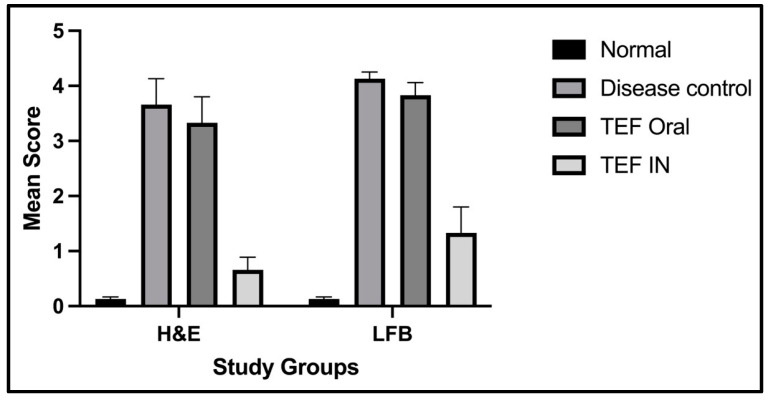
The unbiased semi-quantitative scoring for the histopathological sections where (0) corresponds to no demyelination and inflammation and (5) indicates complete demyelination and inflammation. The IN administered TEF–miR-155-antagomir-loaded NLCs group has exhibited significant (*p* < 0.002) recovery with minimal inflammation and demyelination compared to TEF oral group.

The histochemically stained coronal sections of the bregma regions from the study groups were analyzed and compared (Figure 6 and Figure 7). The H&E-stained normal group sections have established healthy neurons in the CC and CP with a pale and round nucleus, a well-defined nuclear boundary, and prominent nucleoli. Furthermore, the HC and CE, along with their covering meninges and ventricles, have potentially exhibited normal morphology. Subsequently, the LFB-stained sections confirmed that neurons’ normal morphology and myelination were noticed in the HC and CE regions and the midbrain. Conversely, the disease control group displayed numerous degenerated neurons, characteristically with shattered and contracted nuclei in the CE and CP regions. Additionally, multiple foci of necrotic and apoptotic neurons in the CA2 region of HC were detected. Furthermore, multiple foci in CE revealed amyloid plaque deposition. Moreover, the LFB sections revealed regions with potential demyelination in the HC region of the brain and the CA2 region was observed with apoptotic and necrotic neurons. The H&E-stained sections of the TEF oral group have exhibited a higher number of degenerated neurons with fragmented and shrunken nuclei in the CE and CP regions compared to the TEF intranasal treatment group. Similarly, the TEF oral group portrayed multi-focal apoptosis and necrosis in the HC regions, and the CE regions were observed with amyloid plaque deposition. Furthermore, inflammatory cells were noticed proximal to the ventricles of the brain. On the contrary, the TEF intranasal group has not exhibited any apoptotic or necrotic regions of HC and CE. Moreover, normal morphology and myelination of neurons were noticed in CE. The comparisons with the disease group and the TEF oral group revealed a significant reduction in degenerated neurons in the CE and CP regions of the TEF intranasal group. The LFB and H&E-stained coronal sections have potentially highlighted the TEF intranasal group potentiality and have revealed better recovery from the neurodegeneration in the cuprizone-treated animals. These results have established the significance of TEF–miR-155-antagomir-loaded intranasal administration (Figure 8).

The cuprizone-treated disease control group was observed with a severe immunoreactivity of GFAP in the astrocytes and glial cells of the CE region with almost 90% of the glial cells having expressed GFAP protein. However, only a mild immune reactivity of GFAP protein was noted in glial cells of the midbrain with more than 90% of glial cells showing an expression of GFAP protein in the midbrain. Additionally, severe immune reactivity of GFAP protein was observed in glial cells in the region of HC compared to the sections of the normal group with no cuprizone treatment. However, the TEF IN group has exhibited normal immune reactivity of GFAP protein in glial cells of the CE region with less than 25% expression of GFAP protein. Correspondingly, the midbrain region has also exhibited normal immune reactivity with less than 25% GFAP protein expression compared to the TEF oral group and the cuprizone-induced disease group. These results were carefully studied to understand the astrocyte activation.

**Figure 9:**
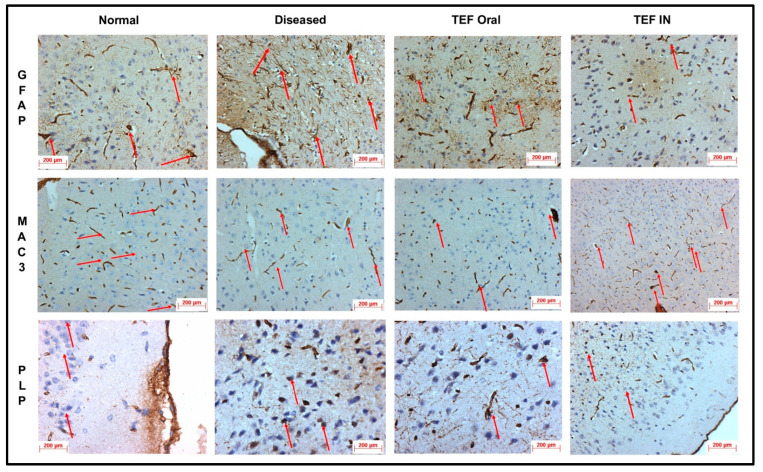
The sections of cerebral cortex. The degree of astrocytosis was measured by IHC of the study groups by GFAP protein immunoreactivity. The macrophages and microglia were measured by IHC of the study groups by MAC3 protein immunoreactivity. The degree of myelin loss was measured by IHC of the study groups by PLP protein immunoreactivity. The images inferred that–IN-administered TEF–miR-155-antagomir-loaded NLCs group has exhibited significant recovery with minimal immunopositive reactivity compared to TEF oral group. (Red arrows indicate immunoreactivity).

**Figure 10:**
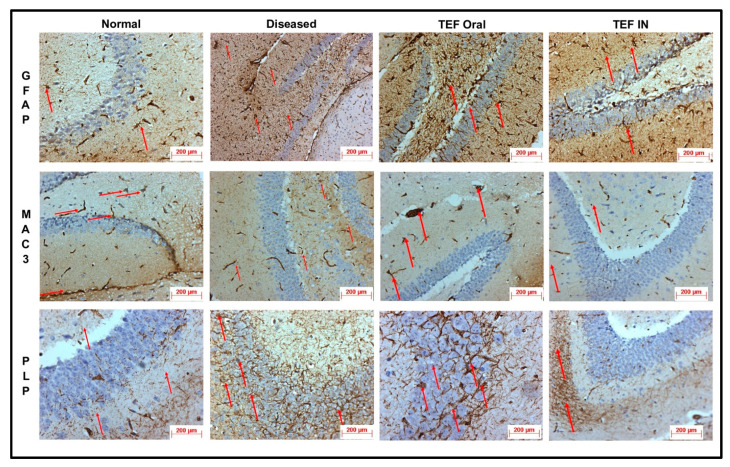
The sections of hippocampus. The degree of astrocytosis was measured by IHC of the study groups by GFAP protein immunoreactivity. The macrophages and microglia were measured by IHC of the study groups by MAC3 protein immunoreactivity. The degree of myelin loss was measured by IHC of the study groups by PLP protein immunoreactivity. The images inferred that–IN-administered TEF–miR-155-antagomir-loaded NLCs group has exhibited significant recovery with minimal immunopositive reactivity compared to TEF oral group. (Red arrows indicate immunoreactivity).

Similarly, the amount of myelin loss was suitably measured by PLP immunoreactivity. The disease control group exhibited severe immune reactivity with glial cells in the midbrain with more than 90% of overexpression. A strong immune reactivity of neuron cells in the region of the hippocampus was noted against the antibodies. Furthermore, strong immunoreactivity was observed in ventricles when compared to the normal group. However, compared to the TEF oral group the TEF–IN group have exhibited potentially minimal immunoreactivity. Furthermore, the microglia and macrophages were measured by MAC3 immunoreactivity. The diseased group with cuprizone has exhibited immune reactivity of MAC3 protein in glial cells of the midbrain and macrophage expression. The HC and CE regions have also exhibited a strong expression of macrophages and MAC3 activity in comparison to the normal group. Conversely, the TEF–IN group have showed encouraging results with minimal MAC3 immunoreactivity compared to the TEF oral group. Figure 9 and Figure 10 exhibit the IHC images.

**Figure 11:**
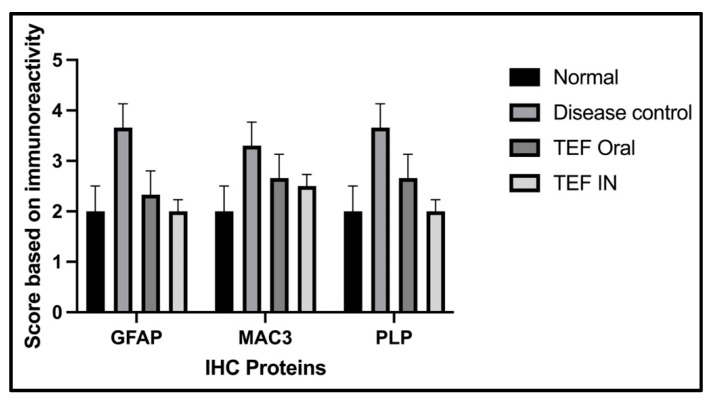
An unbiased semi-quantitative scoring for the immunopositivity of the IHC stained sections of the study groups. The scale shows minimal (0) to severe (5) immunopositivity. The IN-administered TEF–miR-155-antagomir-loaded NLCs group has exhibited significant (*p* < 0.005) recovery with minimal inflammation and demyelination compared to TEF oral group.

The statistical analysis has portrayed higher levels of immunoreactivity levels in the cuprizone-treated disease control group when compared to the normal group (*p* < 0.002). Similarly, the TEF oral group have exhibited significantly higher immunopositive cells compared to the normal group (*p* < 0.002). Fascinatingly, the results show that the IN administration of TEF–miR-155-antagomir-loaded NLCs has effectively reduced the immune-expressing cells compared to the oral group (*p* < 0.005) (Figure 11).

## 4. Conclusions

**Figure 12:**
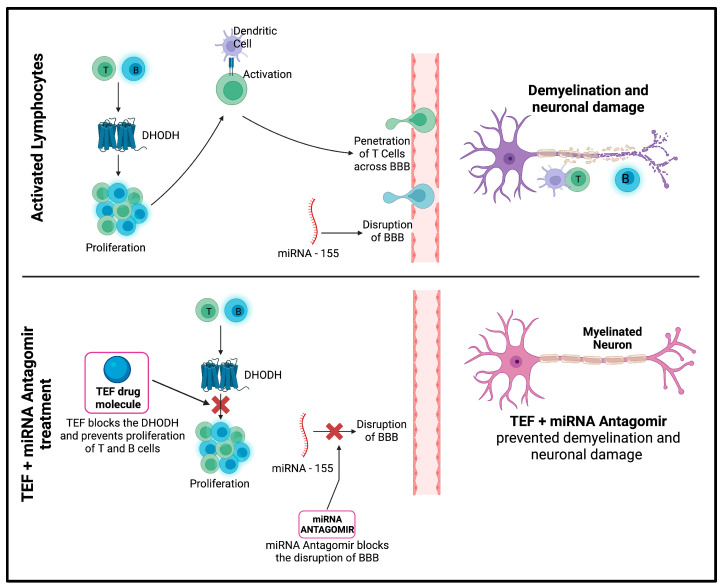
Schematic diagram demonstrating the underlying mechanism highlighting the conclusive evidence of the study.

The study has potentially endorsed successful delivery using IN administration and has achieved higher brain concentrations following IN delivery. The study results suggest that the MS-associated neurodegeneration and demyelination could be managed by combinatorial therapy of miR-155–antagomir and TEF-loaded NLC systems (Figure 12). The loading of miRNAs in nanocarrier systems along with TEF is beneficial in overcoming hindrances encountered by conventional delivery systems, subsequently improving targeting potentials. Additionally, after six weeks of treatment, the NLC systems potentially lowered demyelination in the animal model, indicating the therapeutic potential of this approach in managing MS-associated neurodegeneration and demyelination.

The findings from this study have significant clinical value for managing MS and other neurodegenerative disorders. The research outcome demonstrated the impending use of NLCs to effectively deliver miR-155-antagomir and TEF to the brain via IN administration. This novel delivery method improved targeting potential, bypassed the blood–brain barrier (BBB), and achieved adequate drug levels at the site of action. These results suggest that nanocarrier-loaded therapeutic agents have great potential for safe and economical delivery in treating CNS disorders. RNA-targeting therapies, such as miR-155-antagomir, combined with other agents, such as TEF, could be a promising tool in personalized medicine for the long-term management of neurodegenerative disorders. However, there is no abundant literature on the effective delivery of the miRNAs via intranasal delivery, and, hence, these findings remain instrumental for further studies and the effective delivery of therapeutic molecules via the intranasal route (non-invasive) to CNS in managing neurodegenerative disorders.

This study has shed significant light on the efficient delivery of therapeutic molecules via the IN route, which could be highly beneficial in the management of neurodegenerative disorders. The findings of this study have paved the way for future research in this area, exploring new avenues for the effective treatment of conditions such as multiple sclerosis (MS) and other CNS disorders. The successful implementation of these research findings in clinical practice could potentially revolutionize the management and outcomes of neurodegenerative disorders, bringing about significant improvements in patient care. Overall, this study has contributed valuable insights into the intranasal delivery of therapeutic molecules and its potential applications in the treatment of CNS disorders, highlighting the need for continued research in this area to optimize treatment strategies and improve patient outcomes.

In summary, this study provides important insights into the effective delivery of therapeutic molecules via the IN route for managing neurodegenerative disorders and opens up new avenues for further studies in this area. The successful translation of these findings into clinical practice could significantly improve the management and outcomes of MS and other CNS disorders.

## Figures and Tables

**Table 1:** Pharmacokinetic parameters of developed TEF formulations.

Pharmacokinetic Parameters	Route of Administration and Nature of Formulation					
	NLCs Loaded TEF (IN)		TEF Suspension (IN)		NLCs Loaded TEF (IV)	
	Brain	Plasma	Brain	Plasma	Brain	Plasma
C_max_ (ng/mL)	771.42 ± 17.08	432.44 ± 24.47	679.56 ± 9.08	520.97 ± 9.43	199.25 ± 3.35	880.35 ± 6.58
T_max_ (h)	4	4	2	2	2	2
AUC_0–24 h_ (ng·h/mL)	8229.68 ± 5.50	3309.15 ± 3.79	5998.34 ± 4.59	5032.08 ± 9.45	3086.4 ± 8.16	4436.80 ± 7.55
AUC_0–∞_ (ng·h/mL)	8262.25 ± 6.72	3315.34 ± 6.59	6043.91 ± 8.99	5123.07 ± 8.57	3099.74 ± 11.35	4445.37 ± 11.45
AUMC_0–24_ (ng·h^2^/mL)	61,988.1 ± 6.54	21,447.42 ± 9.67	41,420.44 ± 10.88	32,893.33 ± 8.05	29,844.5 ± 11.62	21,186.20 ± 13.18
AUMC_0–∞_ (ng·h^2^/mL)	62,930.79 ± 21.56	21,604.34 ± 16.46	42,532.69 ± 19.76	35,308.28 ± 23.01	30,177.47 ± 18.55	21,239.19± 12.77
K_el_ (h^−1^)	2.37 ± 0.08	1.93 ± 0.06	1.14 ± 0.06	0.33 ± 0.09	5.64 ± 0.12	4.03 ± 0.13
Relative bioavailability	135.72 ± 10.56					
Absolute Bioavailability	264.9 ± 14.80					

The results were expressed as mean ± SD. The comparisons were considered significant with *p* < 0.05. The NLC IN formulations were significantly different (*p* < 0.002) compared to TEF free drug suspension.

## Data Availability

Not applicable.
